# Leisure Factors Predicting the Happiness of Self-Employed Workers in South Korea

**DOI:** 10.3390/ijerph18189852

**Published:** 2021-09-18

**Authors:** Kwang-Hi Park, Hyunlye Kim, Suin Park

**Affiliations:** 1 College of Nursing, Gachon University, Incheon 21936, Korea; parkkh@gachon.ac.kr; 2 Department of Nursing, College of Medicine, Chosun University, Gwangju 61452, Korea; 3 College of Nursing, Kosin University, Busan 49267, Korea; suinpark@kosin.ac.kr

**Keywords:** employment, happiness, work–life balance, leisure activity

## Abstract

South Korea’s employment status is characterized by a high rate of self-employment and many small-scale self-employed businesses with no employees. This study explored leisure factors relating to self-employed individuals’ subjective happiness based on data from the 2019 National Leisure Activity Survey. The extracted data (N = 2343) were analyzed using descriptive statistics, *t*-test, ANOVA, Pearson’s correlation coefficient, Kendall’s τ_b_ coefficient, Eta correlation coefficient, phi coefficient, and Cramer’s V. And a three-step hierarchical regression analysis was performed to identify multidimensional variables that predict happiness more effectively. In Model 3, which additionally inserted positive and intrinsic factors into Model 2, the explanatory power was significantly increased. The predictors of subjective happiness among self-employed people identified in the final regression model were high economic status (β = 0.05), perceived health status (β = 0.32), financial constraints (β = −0.09), leisure recognition (β = 0.20), and work–life balance (leisure-oriented β = 0.09; work-oriented β = −0.13). This study’s findings will contribute to the establishment of basic data, to prepare empirical measures to improve self-employed individuals’ quality of life.

## 1. Introduction

Happiness can be briefly defined as ‘subjective enjoyment of one’s life as a whole’ and as an evaluative concept in terms of happiness economics [1]. Happiness is highly positively correlated with life satisfaction and the psychological and health domains of quality of life, which are affected by social and environmental aspects of life [2]. It is also closely related to health status, and evidence for the association of happiness with mortality, morbidity, and disease prognosis has been confirmed [3]. According to the World Happiness Report 2021, a landmark survey on global happiness, South Korea ranked 50th out of 156 countries in the world for happiness [4]. However, in a study that compared and analyzed 31 OECD member countries from 1990 to 2017, Korea’s happiness level was found to be in the bottom 30% [5]. It is necessary to explore Koreans’ low happiness index relative to their economic level.

Since most people spend much of their life working, it is inevitable that work plays a major role in shaping their level of happiness [6]. In today’s knowledge-intensive business environment, work-related happiness is a growing topic of study in academia that deserves more attention [7]. Work constitutes an important aspect of happiness, and it is related to work–life balance [8]. Work–life balance as a life value is particularly close to happiness and refers to the relationship between work and non-work aspects of individuals’ lives [9]. Earlier studies have demonstrated that work–life balance is correlated with happiness, both of which positively impact employee performance [8,10]. In the OECD Better Life Index, Korea showed low scores and rankings for work–life balance (4.1 out of 10; 37th out of 40 countries) and life satisfaction (4.0 out of 10; 33rd out of 40) [11]. The present study examines the relationship between work–life balance and happiness in the context of South Korea’s working status.

Previous studies on the relationship between happiness and employment status reported various results, but the results were unclear in the case of the self-employed [12,13,14]. One of the characteristics of Korea’s employment status is the high rate of self-employment. According to OECD Factbook statistics, the self-employment rate in Korea accounted for 24.64 percent of the total employment in 2020 [15]. This is much higher than the average of 15.22 in the European Union’s 27 member states and the seventh highest among the 38 OECD countries. Regarding the status of self-employment, based on Korea’s Economically Active Population Survey, the proportion of small-scale self-employed businesses with no employees is high (75.2% of the self-employed in 2020) and has been increasing in recent years [16]. However, most studies and policies relating to the Korean labor market have focused on wage workers; the present study concerns the happiness experienced directly by the self-employed in Korea under special conditions.

In analyzing the happiness of self-employed people, Warr (2018) investigated whether their working experiences corresponded to personal values with respect to importance [17]. The survey conducted in this study found that self-employed people value self-direction and stimulation in their lives as far more important than those employed in organizations [17]. In Korea’s 2020 national employment trend census, the main motivation for starting self-employment was a desire to run one’s own business, which showed the highest frequency of 71.4% [18]. In a study that analyzed longitudinal data from the German Socio-Economic Panel, shifting from paid work to self-employment helped with job satisfaction, but not life satisfaction, leading to a decrease in leisure satisfaction [19]. This suggests that while self-employment has the advantage of autonomy or self-direction, it can threaten the work–leisure balance and reduce life satisfaction. Therefore, we focused on the perception and experience of leisure life as factors influencing the happiness of the self-employed. 

This study will also look at the general characteristics that predict the happiness of the self-employed. In general, personal, demographic, and socioeconomic factors have been identified through previous studies [14,20,21,22]. In particular, age, marital status, and employment status are known as traditional determinants of happiness [21]. A higher level of education is usually shown to have a positive effect on happiness, but in some cases, it leads to more happiness only if it helps to increase income [14,23]. In the relationship between income and happiness, the Easterlin paradox may emerge, wherein higher income does not lead to greater happiness [24]. 

Previous studies on the happiness, well-being, and leisure lives of self-employed individuals show inconsistent results. A work study conducted in the United States reported that self-employed people reported lower life satisfaction and more health problems, and experienced more positive and negative emotions, than paid employees [25]. A longitudinal study of households in the UK found that freelancers were not less satisfied with life than wage workers; they were much more satisfied with their leisure time and had significantly higher job satisfaction [26]. According to a secondary analysis study based on data from the Korean National Health and Nutrition Examination Survey, self-employed individuals exhibited higher levels of stress, depression, and suicidal ideation than wage workers, and their subjective health status was more negative [27]. In Korea’s National Leisure Activity Survey, the subjective happiness levels and leisure life satisfaction of self-employed workers were reported to be lower than those of full-time workers [28]. In South Korea, studies have investigated leisure-related factors that predict happiness using data from this national report [29,30,31,32], but no study to date has focused on the self-employed. Therefore, the present study aimed to examine the influence of leisure constraints, leisure recognition, and balance between work and leisure on subjective happiness among the self-employed using data from the National Leisure Activities Survey in South Korea. According to global big data studies on the determinants of happiness, it can be classified into factors that contribute to happiness and factors that hinder it [33,34]. This study set the leisure factors that predict happiness in terms of contributors and hindrances. In the current study, hierarchical multiple regression analysis was utilized instead of a single-level model to more effectively identify these multidimensional factors [35].

## 2. Materials and Methods

### 2.1. Design and Data Source

This study is a secondary analysis using national data in South Korea. The data were derived from the 2019 National Leisure Activity Survey published by the Ministry of Culture, Sports, and Tourism (MCST) and conducted by the Korea Culture and Tourism Institute (KCTI) [19]. This nationwide survey was conducted from September to November 2019 using a tablet PC for one-on-one household visit interviews. The data were anonymized and made available to researchers for analysis. We used the original data provided on the KCTI website.

### 2.2. Study Sample

The sample for this study was extracted from data on national leisure activities provided by the MCST. The survey population consisted of citizens aged 15 years or older from 17 cities and provinces nationwide. The sampling frame for this study was established using data from the ‘2017 Population Census’ survey district of the National Statistical Office. Sampling was performed using the stratified multi-stage cluster sampling method. After stratification by region, administrative district, and cluster, 1000 survey districts were systematically extracted, and 10 households were extracted from each survey district. In principle, the survey was conducted on pre-extracted households. If the survey was not possible due to the long-term absence of a member of the household or refusal to respond, the 10th household from the north-right household was selected and replaced from the first sampled household in the same survey district. Data verification was conducted three times during the actual investigation process by phone call, direct evaluation by the supervisor, and computerized program. A supplementary or re-investigation was carried out on the failed questionnaire at each verification stage. In the process of data analysis, time series comparison and verification with the average value of each group (gender, age, region, educational background, occupation, household income, etc.) and previous survey results were made. Non-response adjustment was carried out using the non-response adjustment coefficient and weighting method in units of survey districts. The final sample for this study comprised data from 2343 (23.3%) people who were classified as self-employed, out of a total of 10,060 participants in the survey. This was similar to the proportion (20.6–21.1%) of self-employed people (excluding unpaid family workers among non-wage workers) in 2018–2020 reported in Korea’s Employment Trend Report [18]. However, in the sample of this study, the proportion of self-employed people with employees (14.4%) was quite different from the national indicator (24.8–29.3%) [18]. 

The average age of the sample in this study was 51.94 ± 13.07 years, with more males (61.8%) included. A total of 64.8% had a high school diploma or less. With respect to domestic status, 83.6% were cohabiting with one or more household members, and 73.4% were married. Moreover, 38.5% reported moderate economic status and 37.0% lived in metropolitan cities. The average perceived health status score was 5.15 ± 1.05 out of 1–7. Among the leisure constraints, lack of time showed the highest mean score (5.40 ± 1.40 out of 1–7). The mean score of leisure recognition was 5.34 ± 0.97 out of 1–7. Regarding work–life balance, most participants (45.9%) answered that they were work-oriented. The average score of subjective happiness was 6.86 ± 1.37 out of 1–10. 

### 2.3. Measures

#### 2.3.1. General Characteristics

General characteristics of interest included age, gender, education level, household members living together, marital status, hiring employees, economic status, residential area, and perceived health status. The original response options for education level ranged from ‘elementary school graduate or lower’ (1) to ‘university graduate or higher’ (4), which was simplified to ‘high school graduate or lower’ (1) and ‘university graduate or higher’ (2) in this study. Information on household members living together was collected in the form of actual numbers in the original data but was categorized as ‘yes or no’ in this study. The original options regarding marital status were ‘single’, ‘married’, ‘widowed’, ‘divorced’, and ‘etc.’, and we recoded this as ‘single’, ‘married’, ‘widowed, divorced, etc.’. The options for hiring employees were divided into ‘yes or no’. Economic status was reclassified based on monthly family income. Specifically, less than KRW 3 million (approximately USD 2652) was set as the ‘low’ category, KRW 3 million or more, ~less than 5 million (approximately USD 4420), as the ‘moderate’ category, and KRW 5 million or more as the ‘high’ category. The selection options for the residential area were coded as ‘metropolitan’, ‘small and medium-sized cities’, and ‘rural area’. Perceived health status was set to be rated from ‘very bad’ (1) to ‘very good’ (7).

#### 2.3.2. Leisure-Related Factors

First, the leisure constraints were measured through the question, ‘How much influence do the following constraint factors have on your leisure activities?’ The items for constraint factors were lack of time, financial burden, fine dust, heatwaves or extreme cold, and family health (disease, disability, etc.). Response ratings for each item ranged from 1 (not at all) to 7 (very influential). Second, leisure recognition was measured through the question, ‘Do you think leisure activities are an essential requirement of life?’ Responses ranged from 1 (not at all) to 7 (strongly agree). Third, work–life balance was measured in terms of the concepts of work (education) and leisure life balance. In the original survey, this was defined as a state in which the use of life time of 24 h a day for each, interest and energy for each, and stress from each were not biased toward either side and balanced. The question was, ‘Do you see a good balance between work and leisure in your 24-h life?’ The response scale was 1 (more oriented toward work)—(a balance between work and leisure)—7 (more oriented to leisure). In this study, this variable was reconstructed into three groups. Specifically, groups scoring 1–3 were regarded as work-oriented groups, those scoring 4 as balanced groups, and those scoring 5–7 as leisure-oriented groups.

#### 2.3.3. Subjective Happiness

Subjective happiness levels were measured by the question, ‘How happy do you feel right now?’ Response ratings ranged from 1 (very unhappy) to 10 (very happy). This measurement question on subjective happiness has been used in a number of other secondary analysis studies in Korea using this data source.

### 2.4. Data Analyses

We used IBM SPSS Statistics version 24 (IBM Inc., Armonk, NY, USA) for the data analysis. Descriptive statistics were used to show the distribution or level of general characteristics, leisure-related factors, and subjective happiness. Differences in happiness according to categorical variables were explored through an independent *t*-test and one-way ANOVA with post-hoc Scheffe’s test. Pearson’s correlation coefficient, Kendall’s τ_b_ coefficient, Eta correlation coefficient, phi coefficient, and Cramer’s V were applied to explore the relevance of the variables. Hierarchical multiple regression analysis was performed to identify multidimensional factors predicting happiness. We developed three-step models to investigate the influence of each group of factors on the subjective happiness. Based on the results of prior bivariate analyses, general characteristics and leisure-related factors that showed a significant relationship with happiness were selected as independent variables in the models. Model 1 included general characteristics (age, education level, household member, marital status, hiring employees, economic status, and perceived health status) as independent variables. Model 2 included leisure constraints as a hindrance to happiness (lack of time, financial burden, and family health status) in addition to Model 1. Model 3 included leisure recognition and work–life balance as a contributor to happiness in addition to Model 2. Previously, we tested whether the basic assumptions of the multiple regression analysis model were met. The normality of the residual error was confirmed using a normal probability plot and Kolmogorov–Smirnov test (*p* > 0.05). Using a scatter plot, we found that the assumption of the linearity of the independent and dependent variables was met. Through the examination of the residual scatter plot and Breusch–Pagan test (*p* > 0.05), homoscedasticity was confirmed. The variation inflation factor (VIF) was calculated to test the multicollinearity between independent variables. The range of VIF was 1.14~2.98, and all variables were determined as having no multicollinearity. A *p*-value of < 0.05 (two-sided) was considered statistically significant.

## 3. Results

### 3.1. Relevance of General Characteristics and Leisure-Related Factors for Happiness

Table 1 presents the association of general characteristics and leisure-related factors for happiness by independent *t*-test, ANOVA, and correlation analysis. Age was negatively correlated with happiness (r = −0.11, *p* < 0.001). The subjective happiness score was significantly higher in the group with more than college education (t = −6.39, η = 0.13, *p* < 0.001) and household members (t = −3.75, η = 0.08, *p* < 0.001). Participants who were currently married reported the highest happiness (F = 14.55, η = 0.11, *p* < 0.001). Subjective happiness was significantly higher among participants who hired employees than those who did not hire anyone (t = −1.98, η = 0.04, *p* = 0.047). The subjective happiness was highest in the group who perceived their socio-economic level as high (F = 35.81, τ_b_ = 0.15, *p* < 0.001). Perceived health status was positively correlated with happiness (r = 0.40, *p* < 0.001). Subjective happiness was negatively correlated with time constraints (r = −0.05, *p* = 0.013), financial constraints (r = −0.16, *p* < 0.001), and family health constraints (r = −0.11, *p* < 0.001) among leisure constraints. On the other hand, subjective happiness was positively correlated with leisure recognition (r = 0.32, *p* < 0.001). In terms of work–life balance, the participants who responded as leisure-centered showed the highest happiness score (F = 71.20, η = 0.24, *p* < 0.001), followed by the balanced group and then the work-oriented group. Correlations between independent variables including general characteristics and leisure-related factors are presented in Supplementary Table S1.

### 3.2. Results of Hierarchical Multiple Regression Analysis Predicting Happiness

Table 2 presents the results of the hierarchical multiple regression analysis applied to identify factors predicting happiness. In Model 1, college graduation or higher education level (β = 0.06, *p* = 0.003), high economic status (β = 0.11, *p* < 0.001), and perceived health status (β = 0.39, *p* < 0.001) were predictors of happiness. This model showed explanatory power (R^2^) of 0.18 (*p* < 0.001). In Model 2, financial constraints (β = −0.10, *p* < 0.001) were newly added as a predictor of happiness, and the explanatory power was significantly increased from Model 1 (R^2^ = 0.19, R^2^ change = 0.01, *p* < 0.001). In Model 3, the influence of education level disappeared, and high economic status (β = 0.05, *p* = 0.027), perceived health status (β = 0.32, *p* < 0.001), financial constraints (β = −0.09, *p* < 0.001), leisure recognition (β = 0.20, *p* < 0.001), and work–life balance (leisure-oriented β = 0.09, *p* < 0.001; work-oriented β = −0.13, *p* < 0.001) were identified as predictors of happiness. Model 3’s explanatory power (R^2^) was calculated as 0.27, representing a significant increase from Model 2 (R^2^ change = 0.08, *p* < 0.001).

## 4. Discussion

In this study, self-employed workers’ self-assessed happiness level was 6.86 out of 10, which was higher than daily workers, similar to temporary workers, and lower than regular workers [28]. In comparison to the results of studies using data from other years of the same survey, it was lower than that of leisure sports participants [30] and almost the same as for all subjects, including all types of employment [31]. Based on evidence from the World Happiness Report, the self-employed are generally less happy than full-time employees on a global average [6]. Since the relationship between self-employment and well-being is multifaceted, we explored various related factors and possible socio-environmental contexts that may predict self-employed individuals’ perceived happiness levels.

The general characteristics relating to happiness in this study were age, education level, household members, marital status, hiring employees, economic status, and perceived health status. In other words, the self-employed felt happier when they were younger, had cohabitants or more stable family structures, had a higher economic status, and had a positive perception of their health. These findings have been consistently confirmed in other, previous studies [23,31,32,36,37], and similar results were also found in the study of wage workers [38,39]. This study did not confirm the Easterlin paradox that higher income does not lead to greater happiness [24]. This was similar to the Dutch case and in contrast to the Japanese case in a study comparing the determinants of happiness in Japan and the Netherlands [34]. A formal mechanism of the Easterlin paradox reported recently in China was that with economic growth, material needs upgrade to enjoyment needs, and return to well-being from material conditions decreases [40]. Therefore, it can be interpreted that these mechanisms do not yet work for the self-employed in Korea. Meanwhile, higher happiness levels were also reported by participants who had attended higher education (college graduation or higher). This is consistent with the results of several studies conducted in South Korea [31,32,38,39] but differs from the findings of a self-employed study in the United States [25]. This difference is thought to be related to the socio-cultural context whereby educational background is highly valued in Korean society and recognized as a powerful means of realizing social success [41]. Additionally, higher happiness levels were reported by those who hired employees in the present study. This result is inconsistent with UK freelancers, who reported greater life and leisure satisfaction than those who were self-employed with employees [26]. This difference is interpreted as attributable to the characteristics of self-employment in South Korea, where the proportion of small-scale self-employment is high, and their profits and management stability are low [42]. Considering these results, it is necessary to consider the socio-cultural context holistically, to promote or to evaluate individual happiness.

Leisure factors associated with happiness that were identified through difference and correlation analyses in this study included leisure constraints, recognition of the need for leisure, and work–life balance. First, leisure constraints—such as lack of time, financial burden, and family health—had a negative correlation with subjective happiness. In particular, the degree to which ‘lack of time’ hindered leisure life was 5.40 out of 7, which was the highest among the five leisure constraints. A study on time stress found that self-employed men had lower leisure quality and experienced higher levels of time stress than employed men [43]. Self-employment is often perceived as allowing individuals greater autonomy and more flexibility with respect to their time but, in reality, they appear to suffer from a lack of time and its attendant stress. Future studies should aim to help self-employed individuals to distinguish their work and leisure time and manage their time more efficiently. In this study, we found that greater perceptions of leisure time were associated with higher subjective happiness levels. This is in line with findings from earlier studies showing that the cost and time associated with leisure and leisure satisfaction are positively correlated with subjective happiness [30,31]. These findings suggest that people who value leisure as important attain higher happiness levels by investing more leisure-related time and resources into their lives. Finally, in this study, the group who focused more on leisure in life showed the highest happiness levels. This is consistent with the findings of earlier studies that demonstrated a positive correlation between leisure centrality and happiness level [29]. Considering the results so far, a strategy that minimizes the identified leisure constraints, strengthens awareness around leisure, and improves work–life balance will be beneficial in increasing happiness levels among the self-employed.

This study identified the multidimensional factors predicting happiness through hierarchical regression analysis. When general characteristics, hindrances, and contributors were sequentially input as independent variables of the regression model from Model 1 to Model 3, the explanatory power of the model gradually increased at a statistically significant level. This means that reinforcing contributing factors rather than eliminating or minimizing hindering factors may be a better strategy for promoting happiness. Factors predicting the happiness of self-employed individuals identified in the final model were economic status, perceived health status, financial burden constraints, leisure recognition, and work–life balance. These findings are supported by the results of previous studies that identified factors predicting happiness using the same data source [30,31,32]. In particular, among the identified influencing factors, compared to the work–life balance group, the leisure-oriented group felt happier, and the work-oriented group felt less happy. The concept of work–life balance has subjective characteristics, and so individual perceptions of the ‘balanced state’ may vary [9]. In other words, the current work–life balance perspective includes not only the allocation of physical time and energy invested in work and other life areas but also the individual’s value-oriented aspects. According to a study that reconceptualized work-life balance for the 21st century, achieving a satisfactory work–life balance is normally understood as restricting one side (usually work) to create more time for the other [9]. From this aspect of work–life balance, it was found that the participants in this study experienced a higher level of happiness and attained work–life balance when they were able to engage in leisure activities as time for themselves rather than work, with a strong, realistic purpose. Therefore, it is evident that perceived leisure recognition and work–life balance are more important than physical circumstances or external factors in leading a happy life.

This study is meaningful in that it raises interest in the happiness of the self-employed, a group who have been largely unexplored. In addition, the research findings suggest the role and importance of leisure factors among the determinants of happiness and contribute to a better understanding of the concepts of happiness and work-life balance. However, this study has several limitations and methodological considerations. In this study, there are limitations in identifying causal relationships due to the cross-sectional survey method. Since no information on total contacts or response rates was provided from the data source, the possibility of non-response bias cannot be ruled out. As a result of comparing the characteristics of the self-employed in Korea obtained from national indicators with those of the sample of this study, differences were found, limiting the representativeness. Additionally, since there is a possibility of monomethod bias wherein the relationship between variables measured by the self-report method may be inflated due to the action of common method variance [44,45], more careful interpretation of the results is required. Therefore, it is necessary to apply various design strategies in further studies. Future research should investigate the impact of the social distancing and business restrictions in response to the current COVID-19 pandemic on self-employment and its required responsive changes as a new normal model in the post-COVID-19 era.

## 5. Conclusions

The factors predicting the subjective happiness of self-employed people identified based on South Korean national data were economic status, perceived health status, financial burden constraints, leisure recognition, and work–life balance. These findings suggest that internal awareness that aligns with individual values is more important than external and situational factors in leading a happier life. The study’s findings will contribute to the establishment of basic data, to prepare empirical measures aimed at improving happiness and quality of life among self-employed people.

## Figures and Tables

**Table 1:** Relevance of general characteristics and leisure-related factors for happiness (*N* = 2343).

Variables	n (%)	M ± SD	Subjective Happiness			
			M ± SD	t or F	Correlations	*p*
General characteristics						
Age		51.94 ± 13.07 (range: 18–89)			−0.11 ^†^	<0.001
Gender						
Male	1447 (61.8)		6.85 ± 1.35	−0.52	0.01 ^§^	0.602
Female	896 (38.2)		6.88 ± 1.40			
Education level						
≤High school	1519 (64.8)		6.73 ± 1.37	−6.39	0.13 ^§^	<0.001
≥College	824 (35.2)		7.10 ± 0.32			
Household members						
No	384 (16.4)		6.62 ± 1.39	−3.75	0.08 ^§^	<0.001
Yes	1959 (83.6)		6.91 ± 1.36			
Marital status						
Single ^a^	329 (14.0)		6.84 ± 1.41	14.55	0.11 ^§^	<0.001
Married ^b^	1719 (73.4)		6.93 ± 1.33			(Scheffe a, b > c)
Widowed, divorced, etc. ^c^	295 (12.6)		6.47 ± 1.46			
Hiring employees						
No	2006 (85.6)		6.84 ± 1.37	−1.98	0.04 ^§^	0.047
Yes	337 (14.4)		7.00 ± 1.36			
Economic status						
Low ^a^	750 (32.0)		6.57 ± 1.39	35.81	0.15 ^‡^	<0.001
Moderate ^b^	902 (38.5)		6.85 ± 1.37			(Scheffe c > b > a)
High ^c^	691 (29.5)		7.18 ± 1.26			
Residential area						
Metropolitan ^a^	868 (37.0)		6.88 ± 1.33	0.12	0.01 ^§^	0.884
Small and medium-sized Cities ^b^	674 (28.8)		6.85 ± 1.41			
Rural area ^c^	801 (34.2)		6.85 ± 1.37			
Perceived health status		5.15 ± 1.05			0.40 ^†^	<0.001
Leisure-related factors						
Leisure constraints						
Lack of time		5.40 ± 1.40			−0.05 ^†^	0.013
Financial burden		5.22 ± 1.47			−0.16 ^†^	<0.001
Fine dust		4.71 ± 1.52			0.02 ^†^	0.384
Heatwaves or extreme cold		4.72 ± 1.49			−0.03 ^†^	0.193
Family health		4.65 ± 1.78			−0.11 ^†^	<0.001
Leisure recognition		5.34 ± 0.97			0.32 ^†^	<0.001
Work–life balance						
Leisure-oriented ^a^	508 (21.7)		7.36 ± 1.11	71.20	0.24 ^§^	<0.001
Balanced ^b^	760 (32.4)		6.99 ± 1.29			(Scheffe a > b > c)
Work-oriented ^c^	1075 (45.9)		6.53 ± 1.44			
Dependent variable						
Subjective happiness		6.86 ± 1.37				

M = mean; SD = standard deviation; ^†^ Pearson’s correlation coefficient; ^‡^ Kendall’s τ_b_ coefficient; ^§^ Eta correlation coefficient η. In the variables of Marital status, “a” is the Single category, “b’ is the Married category, and “c” is the Widowed, divorced, etc. category. In the Economic status, “a” is the Low category, “b” is the Moderate category, and “c” is the High category. In the Residential area, “a” is the Metropolitan category, “b” is the Small and medium-sized cities, and “c” is the Rural area category. In the Work–life balance, “a” is the Leisure-oriented category, “b” is the Balanced, and “c” is the Work-oriented category.

**Table 2:** Hierarchical multiple regression analysis predicting happiness (*N* = 2343).

Variables	Categories	Model 1			Model 2			Model 3		
		β	t	*p*	β	t	*p*	β	t	*p*
Age		0.04	1.37	0.169	0.02	0.80	0.421	0.01	0.13	0.740
Education level	≥College	0.06	2.93	0.003	0.06	2.79	0.005	0.03	1.42	0.201
(Ref. ≤ High school)										
Household member	Yes	−0.01	−0.18	0.855	0.01	0.13	0.898	0.01	−0.03	0.828
(Ref. No)										
Marriage	Single	−0.01	−0.13	0.898	−0.01	−0.39	0.693	−0.02	−0.12	0.410
(Ref. Bereavement, divorce, etc.)	Married	0.06	1.83	0.067	0.05	1.51	0.129	0.05	2.28	0.140
Hiring employees	Yes	−0.01	−0.73	0.463	−0.03	−1.28	0.200	−0.01	−0.84	0.823
(Ref. No)										
Economic status	Moderate	0.04	1.68	0.092	0.03	1.42	0.155	0.02	1.21	0.422
(Ref. low)	High	0.11	4.42	<0.001	0.10	4.11	<0.001	0.05	2.57	0.027
Perceived health status		0.39	19.75	<0.001	0.38	19.12	<0.001	0.32	13.62	<0.001
Constraints—lack of time					−0.02	−1.06	0.289	0.01	2.56	0.701
Constraints—financial burden					−0.10	−4.17	<0.001	−0.09	−3.74	<0.001
Constraints—family health status					−0.01	−0.56	0.574	−0.03	−2.75	0.088
Leisure recognition								0.20	7.24	<0.001
Work–life balance	Leisure-oriented							0.09	4.53	<0.001
(Ref. balanced)	Work-oriented							−0.13	−6.09	<0.001
	R^2^	0.18 ***			0.19 ***			0.27 ***		
	ΔR^2^				0.01 ***			0.08 ***		

*** *p* < 0.001; M = mean; SD = standard deviation; Ref. = reference group; Adj. = adjusted.

## Supplementary Materials

The following are available online at https://www.mdpi.com/article/10.3390/ijerph18189852/s1, Table S1: Correlations between independent variables.

## References

[1] Rahman A.A., Veenhoven R. (2018). Freedom and happiness in nations: A research synthesis. Appl. Res. Qual. Life.

[2] Medvedev O.N., Landhuis C.E. (2018). Exploring constructs of well-being, happiness and quality of life. PeerJ.

[3] Steptoe A. (2019). Happiness and health. Annu. Rev. Public Health.

[4] Helliwell J.F., Huang H., Wang S., Norton M., Helliwell J.F., Richard L., Jeffrey S., Jan-Emmanuel D.N. (2021). World happiness, trust and deaths under COVID-19. World Happiness Report 2021.

[5] Park M., Park C. (2020). Study on the happiness of Koreans using a new happiness indicator. Korean Econ. Forum.

[6] De Neve J.-E., Ward G. (2017). Does work make you happy? evidence from the world happiness report. Harv. Bus. Rev..

[7] Salas-Vallina A., Alegre J., Guerrero R.F. (2018). Happiness at work in knowledge-intensive contexts: Opening the research agenda. Eur. Res. Manag. Bus. Econ..

[8] Adnan Bataineh K. (2019). Impact of work-life balance, happiness at work, on employee performance. Int. Bus. Res..

[9] Kelliher C., Richardson J., Boiarintseva G. (2019). All of work? All of life? Reconceptualising work-life balance for the 21st century. Hum. Resour. Manag. J..

[10] Otken A., Erben G.S. (2013). The relationship between work-life balance and happiness from the perspectives of generation X and Y. Humanit. Soc. Sci. Rev..

[11] OECD Better Life Index. https://www.oecdbetterlifeindex.org/countries/korea/.

[12] Mendonca C., Shrivastava A., Pietschnig J. (2020). The effect of adaptive capacity, culture and employment status on happiness among married expatriate women residing in Dubai. Curr. Psychol..

[13] Yagi T., Urakawa K., Yonezaki K., Toshiaki T. (2016). Happiness and employment status. Advances in Happiness Research.

[14] Yakut S.G., Bacaksız N.E., Camkıran C. (2021). Socio-demographic determinants of happiness in Turkey. BMIJ.

[15] OECD iLibrary. https://www.oecd-ilibrary.org/.

[16] Statistics Korea (2021). Economically Active Population Survey: December 2020 and Annual Employment Trends.

[17] Warr P. (2018). Self-employment, personal values, and varieties of happiness–unhappiness. J. Occup. Health Psychol..

[18] Statistics Korea (2020). Economically Active Population Survey: Results of Additional Survey on Non-Wage Working and Economically Inactive Population in August 2020.

[19] van der Zwan P., Hessels J., Rietveld C.A. (2018). Self-employment and satisfaction with life, work, and leisure. J. Econ. Psychol..

[20] Azizi M., Mohamadian F., Ghajarieah M., Direkvand-Moghadam A. (2017). The effect of individual factors, socioeconomic and social participation on individual happiness: A cross-sectional study. J. Clin. Diagn. Res..

[21] Cordero J.M., Salinas-Jiménez J., Salinas-Jiménez M.M. (2017). Exploring factors affecting the level of happiness across countries: A conditional robust nonparametric frontier analysis. Eur. J. Oper. Res..

[22] Sharifzadeh H., Mirmohammad Taber S., Adlipour S. (2018). A study of factors affecting social happiness in Iran: A meta-analysis of conducted research. J. Strategy Cult..

[23] Eren K.A., Aşıcı A.A. (2017). The determinants of happiness in Turkey: Evidence from city-level data. J. Happiness Stud..

[24] Angeles L. (2011). A closer look at the Easterlin paradox. J. Socio-Econ..

[25] Bencsik P., Chuluun T. (2021). Comparative well-being of the self-employed and paid employees in the USA. Small Bus. Econ..

[26] van der Zwan P., Hessels J., Burger M. (2020). Happy free willies? Investigating the relationship between freelancing and subjective well-being. Small Bus. Econ..

[27] Kwon M., Kim S. (2019). Convergence factors affecting quality of life of wage worker and self-employed. J. Korea Converg. Soc..

[28] Ministry of Culture, Sports and Tourism (2019). 2019 National Leisure Activity Survey.

[29] Nam Y.J., Oh S.J., Kim E.H., Nam S.K. (2018). The effects of stress and leisure activities on satisfaction and happiness of employees. AJMAHS.

[30] Lee Y., Hwang S. (2019). The determinants of happiness in participants of leisure sports: Hierarchical regression analysis. KSLRP.

[31] Lee Y., Hwang S., Kim C. (2020). Leisure resource factors affecting happiness. J. Leis. Stud..

[32] Lee M., Hong Y., Yoon K. (2016). Impacts of leisure activities on individual happiness: Focusing on the mediating effects of leisure satisfaction. J. Cult. Policy.

[33] Jaswal V., Kishore K., Muniraju M., Jaswal N., Kapoor R. (2020). Understanding the determinants of happiness through Gallup World Poll. J. Fam. Med. Prim. Care.

[34] Takahashi Y., Fukushima S., Hagiwara R. (2018). Determinants of happiness in Japan and the Netherlands: Macro and micro analysis and comparison. Asia-Pac. Rev..

[35] Van Dusen B., Nissen J. (2019). Modernizing use of regression models in physics education research: A review of hierarchical linear modeling. Phys. Rev. Phys. Educ. Res..

[36] Tanzer J.R. (2021). Developing authentic happiness: Growth curve models to assess lifelong happiness. J. Posit Psychol..

[37] Oshio T., Kobayashi M. (2010). Income inequality, perceived happiness, and self-rated health: Evidence from nationwide surveys in Japan. Soc. Sci. Med..

[38] Lee M.Y., Lee H.J., An S.G., Yoon S., Choi S.L., Yoon S.T. (2019). The effects of wage workers’ work and leisure on happiness. Q. J. Labor Policy.

[39] Yoon S., Lee H.J. (2020). A study on factors affecting happiness of wage workers. Korean J. Soc. Welf..

[40] Li L., Shi L. (2019). Economic growth and subjective well-being: Analyzing the formative mechanism of Easterlin Paradox. J. Chin. Sociol..

[41] Kang C.D. (2019). A study on the social phenomenon of Haglyeg perversion in Korea from Lacan’s perspective. Korea Educ. Rev..

[42] Shin D.J., Choi P.K. (2018). The cause of the increase of small self-employed in Korea: The relationship between deindustrialization and small self-employed. JIEB.

[43] Gimenez-Nadal J.I., Ortega-Lapiedra R. (2010). Self-employment and time stress: The effect of leisure quality. Appl. Econ. Lett..

[44] Podsakoff P.M., MacKenzie S.B., Lee J.Y., Podsakoff N.P. (2003). Common method biases in behavioral research: A critical review of the literature and recommended remedies. J. Appl. Psychol..

[45] Spector P.E. (2006). Method variance in organizational research: Truth or urban legend?. Organ. Res. Methods.

