# Supplementary material for: Leisure Factors Predicting the Happiness of Self-Employed Workers in South Korea
Source: Int J Environ Res Public Health. 2021 Sep 18;18(18):9852. doi: 10.3390/ijerph18189852 (PMC8470773; doi:10.3390/ijerph18189852)
Supplement: Supplementary file 1 [file ijerph-18-09852-s001.zip › ijerph-1336175-supplementary.pdf]

Supplementary Table S1. Correlations between independent variables.

Variables	1.	2.	3.	4.	5.	6.	7.	8.	9.	10.	11.	12.	13.	14.	15.
	**r, Kendall's τ_b, η, ϕ, or Cramer's V (*p*)**														
1. Age	-														
2. Gender	0.04§	-													
	(0.075)														
3. Education level	0.46§	-0.07¶	-												
	(<0.001)	(0.001)													
4. Household members	0.03§	-0.08¶	0.03¶	-											
	(0.147)	(<0.001)	(0.159)												
5. Marital status	0.52§	0.17‖	0.22‖	0.67‖	-										
	(<0.001)	(<0.001)	(<0.001)	(<0.001)											
6. Hiring employees	0.16§	-0.14¶	0.21¶	0.00¶	0.09‖	-									
	(<0.001)	(<0.001)	(<0.001)	(0.971)	(<0.001)										
7. Economic status	-0.26‡	0.04§	0.28§	0.29§	0.24§	0.29§	-								
	(<0.001)	(0.051)	(<0.001)	(<0.001)	(<0.001)	(<0.001)									
8. Residential area	0.17§	0.03‖	0.19‖	0.03‖	0.09‖	0.08‖	0.27§	-							
	(<0.001)	(0.402)	(<0.001)	(0.315)	(<0.001)	(0.001)	(<0.001)								
9. Perceived health status	-0.28†	0.10§	0.16§	0.04§	0.16§	0.05§	0.14‡	0.04§	-						
	(<0.001)	(<0.001)	(<0.001)	(0.059)	(<0.001)	(0.018)	(<0.001)	(0.086)							
10. Constraints – lack of time	-0.14†	0.02§	0.01§	0.05§	0.04§	0.04§	0.02‡	0.09§	0.05†	-					
	(<0.001)	(0.307)	(0.611)	(0.012)	(0.075)	(0.058)	(0.273)	(<0.001)	(0.028)						
11. Constraints – financial burden	-0.02†	0.05§	0.04§	0.00§	0.06§	0.11§	-0.08‡	0.07§	-0.10†	0.46†	-				
	(0.470)	(0.024)	(0.049)	(0.913)	(0.018)	(<0.001)	(<0.001)	(0.004)	(<0.001)	(<0.001)					

12. Constraints - fine dust	-0.05†	0.04§	0.06§	0.05§	0.02§	0.04§	0.04‡	0.09§	-0.03†	0.31†	0.45†	-			
	(0.018)	(0.081)	(0.002)	(0.023)	(0.769)	(0.035)	(0.013)	(<0.001)	(0.136)	(<0.001)	(<0.001)				
13. Constraints - Heatwaves or extreme cold	0.01†	0.03§	0.03§	0.03§	0.04§	0.04§	0.02‡	0.09§	-0.02†	0.30†	0.40†	0.76†	-		
	(0.813)	(0.195)	(0.131)	(0.174)	(0.120)	(0.078)	(0.216)	(<0.001)	(0.248)	(<0.001)	(<0.001)	(<0.001)			
14. Constraints - Family health	0.01†	0.04§	0.02§	0.02§	0.03§	0.04§	-0.02‡	0.02§	-0.12†	0.27†	0.43†	0.48†	0.57†	-	
	(0.734)	(0.075)	(0.377)	(0.388)	(0.341)	(0.091)	(0.396)	(0.563)	(<0.001)	(<0.001)	(<0.001)	(<0.001)	(<.001)		
15. Leisure recognition	-0.14†	0.03§	0.15§	0.05§	0.08§	0.01§	0.14‡	0.14§	0.27†	0.11†	-0.04†	0.02†	0.02†	-0.07†	-
	(<0.001)	(0.178)	(<0.001)	(0.009)	(<0.001)	(0.556)	(<0.001)	(<0.001)	(<0.001)	(<0.001)	(0.056)	(0.239)	(0.383)	(0.001)	
16. Work-life balance	0.03§	0.05¦¦	0.10¦¦	0.03¦¦	0.04¦¦	0.01¦¦	0.12§	0.11¦¦	0.07§	0.26§	0.07§	0.09§	0.06§	0.10§	0.11§
	(0.460)	(0.046)	(<0.001)	(0.274)	(0.096)	(0.892)	(<0.001)	(<0.001)	(0.006)	(<0.001)	(0.002)	(<0.001)	(0.016)	(<0.001)	(<0.001)

†Pearson's correlation coefficient; ‡Kendall's τ_b coefficient; §Eta correlation coefficient η; ¶phi coefficient ϕ; ¦¦Cramer's V.
